# Tools for pathogen genetic surveillance: Lessons from the ash dieback invasion of Europe

**DOI:** 10.1371/journal.ppat.1012182

**Published:** 2024-05-23

**Authors:** Jessica A. Peers, Richard M. Leggett, Matthew D. Clark, Mark McMullan

**Affiliations:** 1 The Earlham Institute, Norwich, United Kingdom; 2 Department of Science, The Natural History Museum, London, United Kingdom; Shanghai Center for Plant Stress Biology, CHINA

## Introduction

Human movement and climate change are exposing new environments to pathogens and increasing the rate of invasions [1]. Presently, most pathogens are studied independently, requiring species-specific expertise, particularly at the sampling stage. Genomic surveillance strategies can be applied to identify species presence/absence but also within-species diversity, which may not currently be capitalised upon. Here, we use the ash dieback invasion of Europe [2] to frame a discussion on the significance of different types of genomic variation to invasion biology and review the application of developing microbiome sequencing technologies to pathogen surveillance.

## What is ash dieback?

European ash (*Fraxinus excelsior*) is prevalent throughout Europe; for example, it is the third most common tree species in the United Kingdom. Ash is a keystone species with approximately 1,000 associated species and over 100 obligately/highly associated species [3]. The ash dieback invasion of Europe (see review [4]), caused by *Hymenoscyphus fraxineus*, was first identified in Estonia, 1992, and has since spread west, reaching the UK in 2012 [2]. In its native range of East Asia, *H*. *fraxineus* is a saprophyte with little effect on its host, *F*. *mandshurica* [5]. In Europe, *H*. *fraxineus* causes crown dieback and death of at least 2 ash species [2]. Ash dieback is estimated to cost the UK economy £7.6 billion by 2029 [6].

Slow spread of disease symptoms [7], environmental conditions [8], varying disease resistance [9], and propagule pressure and spread [10] make estimating ash mortality rates difficult. Best estimates suggest mortality of 70% to 85% [7]. Models of plant defence against pathogens focus on resistance genes evolved to recognise different pathogen gene products and trigger immunity [11]. Reduced susceptibility in European ash appears to be mediated by many genes [9], meaning that both natural selection and breeding programmes have a lot of work to do to bring all of those genes together [12].

*H*. *fraxineus* was spread within Europe by trade of infected samplings and by wind and consequently, may be found 50 to 100 km ahead of the infection front [13]. Management of diseases at these scales is difficult, so our attention turns to introduction and reintroduction. Surveillance measures could be important to identify an invasion early or prevent subsequent invasions of genetic diversity by focussing effort on limiting exchanges between reservoirs of diversity, which is critically important for pathogen adaptation.

## How do we investigate intraspecific genomic variation?

Advancing genomic technologies are particularly tractable for the identification of emergent pathogens, especially in cases where culturing requires species-specific knowledge. Sequence-based methods can also be used to understand pathogen populations: to identify sources and important genomic regions, to inform management strategies to reduce transfers between reservoirs of diversity (e.g., [14,15]). Under investigated but equally important, we outline how genomic surveillance can identify haplotypic and structural variation which also facilitate introduction of novel diversity.

### Genetic diversity

**Fig 1:**
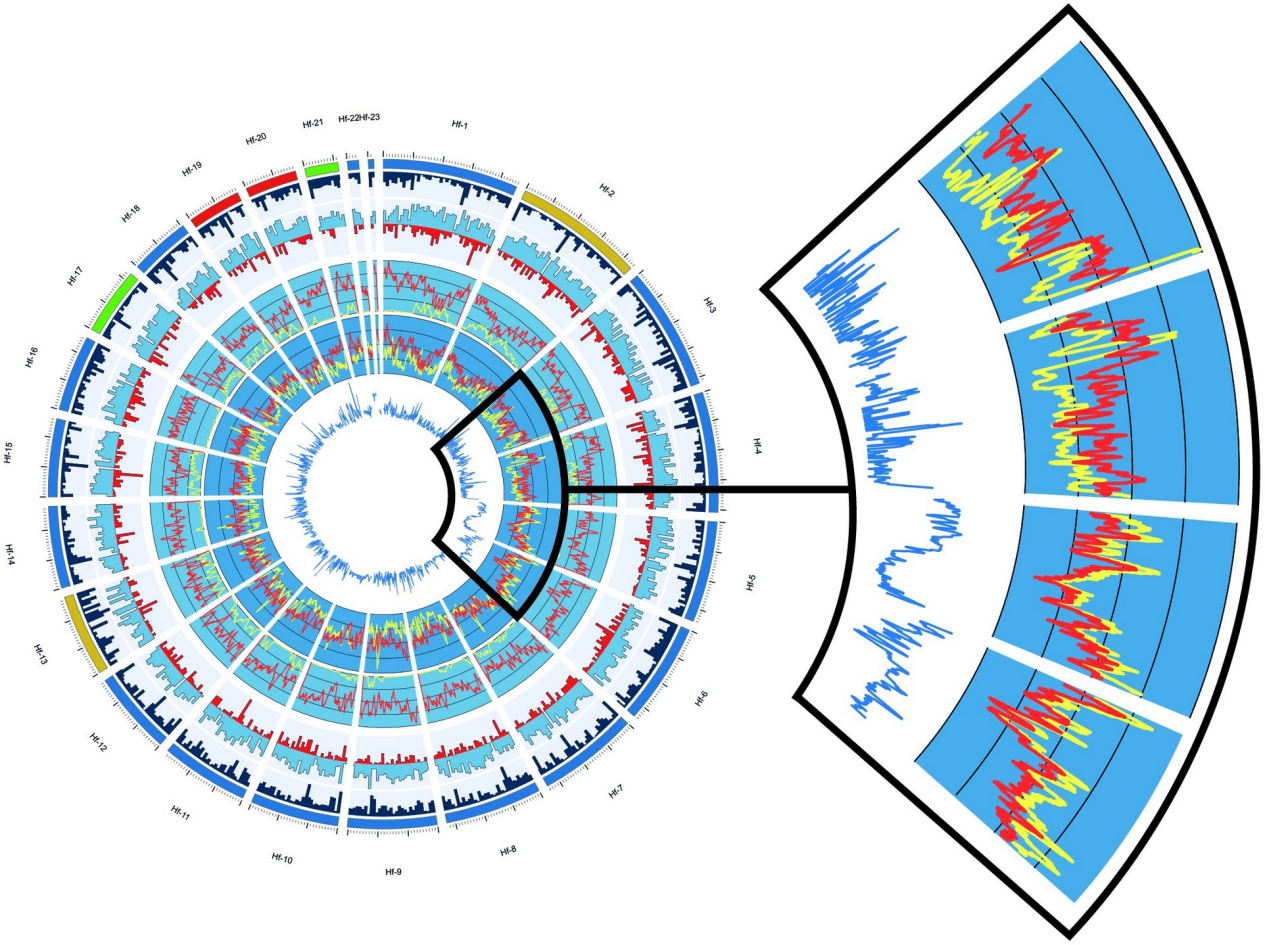
Evidence for preservation of genetic diversity by structural genomic variation in *H*. *fraxineus*. Evidence for duplicated content is replotted against data originally analysed (modified) from McMullan and colleagues [18]. Moving outwards from the centre, tracks detail: cumulative duplication among European invasion isolates, SNP density, nucleotide diversity, gene (blue) and effector (red) density, and repeat density. The exploded section shows for 4 scaffolds, duplicated content, and SNP density for native (red) and invasive (yellow) *H*. *fraxineus* populations. The exploded top 2 scaffolds are representative of the genome-wide pattern, in which SNP density is, as expected, lower in the invasion population alongside sparse duplication, rarely overlapping among individuals. The lower 2 (exploded) scaffolds show that invasion SNP density mirrors that of the native, and duplications overlap, and are shared among individuals throughout the scaffold.

Nucleotide substitutions can identify novel species, perhaps notable where invaders are related to a known pathogen or commensal species (e.g., *Hymenoscyphus* species [16]). However, the level of genetic variation within a species is often measured using the number of single nucleotide polymorphisms (SNPs), which are captured by predominant genome sequencing strategies (Table 1). Nucleotide diversity is an important metric (based on SNP number and frequency) that is determined by the effective population size (and mutation rate). These metrics describe a population’s ability to respond to selection relative to drift [17]. Low numbers of invaders/spores mean that many pathogen introductions are associated with reductions in genetic diversity, as is the case for the ash dieback pathogen (Fig 1; [18]). At the genome scale, the native genetic diversity of an invading pathogen represents a reservoir available for subsequent invasions [19]. At the gene scale, that reservoir of diversity may be particularly stark at effector genes [18], which putatively interact with the host and may modulate defence [20]. Finally, genetic diversity and differentiation among native populations provides valuable information on the biology of the pathogen (e.g., population size and migration rate), which is often overlooked.

**Table 1:** Methods for genomic surveillance and their advantages and disadvantages. Targeted resequencing: Select loci (e.g., genes, SNPs) based on existing data. Resequencing: Sequence whole genomes to map to a reference. Pangenomics: Sequence whole genomes from multiple individuals of the same species and analyse using pangenome-graphs.

Advantages	Disadvantages
**Targeted resequencing/enrichment sequencing** [21]	
Highly targeted SNP diversity All output is relevant Standardisable analysis pipeline Lower cost/increased sample capacity	Reduced representation Potential ascertainment and reference bias Requires target development Structural and haplotypic polymorphism absent
**Whole genome resequencing** [18]	
Genome-wide SNP diversity Minimal ascertainment bias	More expensive than targeted methods Structural and haplotypic polymorphism lacking
**Pangenomics** [22]	
Genome-wide SNP diversity Recovers structural and haplotypic polymorphism Recovers novel (nonreference) genome sequence	More expensive than above methods Assembly and analysis tools still in development

### Haplotype diversity

SNP diversity, identified using targeted/enrichment sequencing, or resequencing in diploids, overlooks haplotype diversity and the impact of recombination. Haplotypes are physically linked combinations of SNPs that are coinherited and broken down over time by recombination. In many fungi, the somatic tissues are haploid and the power to detect the influence of recombination increases as assembly haplotypic contiguity increases, from targeted methods to pangenomics (see Table 1). At the species level, hybridisation recombines divergent genetic diversity and has been associated with host jumps [23] and increased pathogenicity, e.g., Dutch elm disease [15], and is visible as introgression [14].

The costs and benefits of sex are wide ranging (and out of scope), but the potential fitness gains of combining favourable variants and/or the removal of unfavourable ones are greater in heterogeneous and/or novel environments [24]. Evidence for recombination is key to determining the impact of further genetic invasion because recombined genotypes may be more devastating than the sum of their parts. Linkage analysis in the *H*. *fraxineus* invasion shows that recombination does operate, increasing the potential of any further introduced polymorphism [18]. Progress in our understanding of the impact of recombination in pathogen invasion may be made by studying pathogen invasions of crops from wild crop relatives (e.g., [14,25]).

### Structural genomic variation

Perhaps most difficult to detect without pangenomic methods, structural genomic variation (e.g., gene/chromosome duplication, accessory chromosomes, fusions, and inversions) can introduce variation at the haploid genome level [26]. Polymorphism between duplicated regions (or genes) on a given haplotype is not lost during an invasion bottleneck in the same way that polymorphism between alleles is. Given the higher quality of the genomes required to measure this kind of polymorphism, these processes may be currently underestimated [27].

Fungi appear particularly adept at gain, loss, inversion, and fusion of regions of their genome [28–30]. There is evidence in *H*. *fraxineus* for the preservation of genetic diversity across regions of the invaders’ genome (Fig 1). However, this evidence comes from short-read resequencing, which is not adequate to address these questions (Fig 1). Depauperate native range sampling and a lack of long read assemblies (or pangenome) mean the mechanism for this preservation is uncertain: Is this duplication preserved across the native range (i.e., duplication occurred once in microevolutionary history), or is there polymorphism among individuals (i.e., ongoing duplication)? These questions are particularly important to address for invaders because they establish a propensity for recurrent successful invasions despite bottlenecks.

## How can we surveil for pathogens?

Targeted resequencing takes advantage of available sequence data, while whole genome resequencing increases diversity resolution without prior data except a reference genome. However, as sequencing costs are reducing, pangenomics is more attractive for the most complete information. Our understanding of pathogen invaders may be increased further if we consider sampling environments as opposed to distinct species sequencing efforts.

Pathogen capture methodologies must vary with the medium in question (e.g., air, soil, water). Here, we have considered genomic surveillance strategies employed to sample the air, in part because of the propensity of *H*. *fraxineus* to spread by air [13]. However, strategies that describe diversity of other mediums may be even more beneficial, given our increased ability to manage soil systems compared to air, for example.

Air sequencing circumvents the requirement for species-specific expertise to identify an invasion and collect samples in a timely fashion and involves capturing biological particles from the air, extracting DNA, library preparation, and sequencing. This technology is in its infancy but could precede current approaches for identifying invasions [31]. In future, air sequencing could provide rapid population diversity data with sequence reads long enough to determine structural/haplotypic variation in a pathogen agnostic way.

Air sequencing approaches have been used to measure biodiversity [32,33] and to characterise seasonal changes in bacteria and fungi in urban environments [34]. Recovering large amounts of DNA from aerosol samples is challenging, as is identifying their source given the incompleteness of public databases. However, whole genome shotgun approaches have also demonstrated usefulness in clinical [35] and agricultural environments. In a mixed crop field, wheat rust (*Puccinia striiformis f*. *sp*. *tritici*) can be identified to strain level, in and among pathogens specific to the other crops found there [36]. The ability to identify strain specific differences is critical for population genomic surveillance, e.g., COG-UK consortium, which tracked COVID in the UK. Furthermore, DNA can be recovered and sequenced from existing filters used in air monitoring networks [37], providing a cost-effective mechanism for data collection from before the alarm was raised.

## Conclusions

When considering the efficacy of genomic surveillance, it is important to understand the invasive and native genetic structure of pathogens as well as the biology each method is tuned to detect. Until relatively recently, the focus has been on understanding genetic diversity (i.e., SNPs), but haplotype and structural genetic variation can alleviate and circumvent reductions in adaptive diversity brought about by invasion bottlenecks. Here, we highlight the benefit and feasibility of surveilling for evidence of recombination and structural diversity. Existing genomic technologies already provide this level of information, yet they require species-specific expertise to sample. The devastation of the ash dieback pathogen, *H*. *fraxineus*, and increasing invasions of species due to climate change elevate the importance of pathogen-agnostic surveillance. Improved bioinformatics tools, open-data practices, and global resources for pathogen identification could be invaluable to identify subsequent genetic invasions in existing invasion ranges, as well as invasion routes and primary invasions in novel environments.

## Methods

Data, originally published in McMullan and colleagues’ article [18], was reanalysed here using CNVnator [38]. CNVnator uses short read depth data to identify evidence for duplication (α = 0.05) and was run for each European isolate. Duplication regions were plotted as a cumulation of events among all European isolates (Fig 1).
